# Diabetic Foot Australia guideline on footwear for people with diabetes

**DOI:** 10.1186/s13047-017-0244-z

**Published:** 2018-01-15

**Authors:** Jaap J. van Netten, Peter A. Lazzarini, David G. Armstrong, Sicco A. Bus, Robert Fitridge, Keith Harding, Ewan Kinnear, Matthew Malone, Hylton B. Menz, Byron M. Perrin, Klaas Postema, Jenny Prentice, Karl-Heinz Schott, Paul R. Wraight

**Affiliations:** 1 School of Clinical Sciences, Queensland University of Technology, Brisbane, QLD Australia; 2 Diabetic Foot Australia, Brisbane, QLD Australia; 3 Wound Management Innovation Cooperative Research Centre, Brisbane, QLD Australia; 4 Allied Health Research Collaborative, Metro North Hospital & Health Service, Brisbane, QLD Australia; 5 Southern Arizona Limb Salvage Alliance (SALSA), Department of Surgery, University of Arizona College of Medicine, Tucson, AZ USA; 6 Department of Rehabilitation, Academic Medical Center, University of Amsterdam, Amsterdam Movement Sciences, Amsterdam, the Netherlands; 7 Vascular Surgery, The University of Adelaide, Adelaide, South Australia Australia; 8 University Dean of Clinical Innovation, Professor of Wound Healing Research, Cardiff University, Cardiff, UK; 9 High Risk Foot Service, Liverpool Hospital, South Western Sydney Local Health District, Sydney, NSW Australia; 10 Discipline of Podiatry, School of Allied Health, College of Science, Health and Engineering, La Trobe University, Bundoora, VIC Australia; 11 La Trobe Rural Health School, College of Science, Health and Engineering, La Trobe University, Bendigo, VIC Australia; 12 Department of Rehabilitation Medicine, University of Groningen, University Medical Center Groningen, Department of Rehabilitation Medicine, Groningen, the Netherlands; 13 Wound Consultant, Trojan Health, Perth, WA Australia; 14 School of Health and Human Sciences (Pedorthics) Southern Cross University Gold Coast Campus, Bilinga, QLD Australia; 15 Diabetic Foot Unit, Royal Melbourne Hospital, Melbourne, VIC Australia

**Keywords:** Foot ulcer, Diabetes mellitus, Footwear, Prevention, Guideline

## Abstract

**Background:**

The aim of this paper was to create an updated Australian guideline on footwear for people with diabetes.

**Methods:**

We reviewed new footwear publications, (inter)national guidelines, and consensus expert opinion alongside the 2013 Australian footwear guideline to formulate updated recommendations.

**Result:**

We recommend health professionals managing people with diabetes should: (1) Advise people with diabetes to wear footwear that fits, protects and accommodates the shape of their feet. (2) Advise people with diabetes to always wear socks within their footwear, in order to reduce shear and friction. (3) Educate people with diabetes, their relatives and caregivers on the importance of wearing appropriate footwear to prevent foot ulceration. (4) Instruct people with diabetes at intermediate- or high-risk of foot ulceration to obtain footwear from an appropriately trained professional to ensure it fits, protects and accommodates the shape of their feet. (5) Motivate people with diabetes at intermediate- or high-risk of foot ulceration to wear their footwear at all times, both indoors and outdoors. (6) Motivate people with diabetes at intermediate- or high-risk of foot ulceration (or their relatives and caregivers) to check their footwear, each time before wearing, to ensure that there are no foreign objects in, or penetrating, the footwear; and check their feet, each time their footwear is removed, to ensure there are no signs of abnormal pressure, trauma or ulceration. (7) For people with a foot deformity or pre-ulcerative lesion, consider prescribing medical grade footwear, which may include custom-made in-shoe orthoses or insoles. (8) For people with a healed plantar foot ulcer, prescribe medical grade footwear with custom-made in-shoe orthoses or insoles with a demonstrated plantar pressure relieving effect at high-risk areas. (9) Review prescribed footwear every three months to ensure it still fits adequately, protects, and supports the foot. (10) For people with a plantar diabetic foot ulcer, footwear is not specifically recommended for treatment; prescribe appropriate offloading devices to heal these ulcers.

**Conclusions:**

This guideline contains 10 key recommendations to guide health professionals in selecting the most appropriate footwear to meet the specific foot risk needs of an individual with diabetes.

## Background

Diabetic foot ulcers are a costly complication of diabetes, reducing people’s quality of life, and increasing morbidity, mortality and healthcare expenditure [1–4]. The annual incidence of foot ulcers in people with diabetes is approximately 2%, both globally [3] and in Australia [5], and the lifetime risk is between 19% and 34% [6]. Additionally, diabetic foot ulcers are the leading cause of lower extremity amputations and cause approximately 2% of all hospitalisations [3–5, 7, 8]. Therefore, the prevention of diabetic foot ulcers is of paramount importance.

Diabetic foot ulcers are typically caused by repetitive stresses (shear and pressure) on the foot in the presence of the diabetes-related complications of peripheral neuropathy or peripheral artery disease, and their healing is often complicated by the development of infection [9–13]. Use of inappropriate footwear or walking barefoot typically increases the magnitude of the local mechanical repetitive stresses on the foot that are leading causes of the development of diabetic foot ulceration [9–12]. Thus, it is recommended that people with diabetes wear appropriate footwear designed to reduce repetitive stresses at all times, to help prevent diabetic foot ulceration [14, 15].

In 2013, the Australian Diabetes Foot Network published one of the first nationwide practical guidelines on the provision of footwear for people with diabetes [16]. Since this publication, pivotal new studies [9, 17–30] and international guidelines [10, 14, 15] have been published on footwear for people with diabetes. This new literature provides a stronger evidence-base for the effectiveness of footwear in ulcer prevention for people with diabetes, new data-driven directions for the prescription of footwear, and new evidence on the importance of adherence to wearing footwear [9, 10, 14, 15, 17–30]. The aim of this article is to update the 2013 Australian practical guideline [16], and thereby creating a new *Diabetic Foot Australia guideline on footwear for people with diabetes*.

## Methods

### Procedure for developing the guideline

The *Diabetic Foot Australia guideline on footwear for people with diabetes* aims to provide guidance to the multidisciplinary healthcare professionals involved in the provision of footwear for people with diabetes. The *Australian Diabetes Foot Network 2013 practical guideline on the provision of footwear* [16] was used as a baseline for the creation of this updated guideline. Information from the 2013 footwear guideline was updated first by the primary author after reviewing and incorporating any new footwear-related recommendations from the most recent Australian National Health and Medical Research Council (NHMRC) diabetic foot guideline [31] and the International Working Group on the Diabetic Foot (IWGDF) guidance documents [10, 14, 15]. The primary author then reviewed and incorporated common findings from all recent systematic reviews on footwear interventions for people with diabetes [17–22], recent randomized controlled trials included in these reviews [23, 24], and finally any further studies obtained from hand searching reference lists of these articles and an additional non-systematic search of the literature [9, 25–30]. After collating all findings, three tables and one figure were created describing footwear requirements and offloading effects of footwear modifications to prevent diabetic foot ulceration, based on published literature [10, 15, 16, 32–34] and expert opinion. Furthermore, to ensure a common and clear vocabulary between all the different multidisciplinary healthcare professionals involved in the provision of footwear for people with diabetes [14, 31], a table of definitions for common terms related to footwear for people with diabetes was developed based on literature [15, 16, 32, 33] and expert opinion (Table 1).

**Table 1:** List of definitions related to footwear for people with diabetes

Term	Definition
Abnormal foot shape	A foot shape that cannot be accommodated in pre-fabricated footwear. This includes, but is not limited to, feet with: hallux valgus, clawed/hammer toes, severe pes-planus or cavus foot type, abnormally wide feet, flat foot, minor amputation or Charcot foot.
Bespoke footwear	Synonym for “Custom-made medical grade footwear”.
Custom-made footwear	Synonym for “Custom-made medical grade footwear”.
Custom-made insole	An insole that is custom-made to the individual’s foot using a 2D or 3D impression of the foot, and that is often built-up in a multi-layer construction. This may also incorporate other features, such as a metatarsal pad or metatarsal bar. The insole is designed to conform to the shape of the foot, providing cushioning and redistribution of plantar pressure.
Custom-made medical grade footwear	Footwear uniquely manufactured for one person, when this person cannot be safely accommodated in pre-fabricated medical grade footwear. It is made to accommodate deformity and relieve pressure over at-risk sites on the plantar and dorsal surfaces of the foot. In-depth assessment, multiple measurements, impressions or a mould, and a positive model of a person’s foot and ankle are generally required for manufacture.
Customised insole	Term to denote a pre-fabricated insole to which minor modifications specific to a person’s foot may have been made. This term is not synonymous with “Custom-made insole”.
Depth-inlay footwear	Synonym for Extra-depth footwear.
Depth footwear	Synonym for Extra-depth footwear.
Extra-depth footwear	Footwear constructed with additional depth and volume in order to accommodate deformity such as claw/hammer toes and/or to allow for space for a thick insole. Usually a minimum of 5 mm (~3/16″) depth is added compared to pre-fabricated footwear. Even greater depth is sometimes provided in footwear that is referred to as double depth or super extra-depth.
Footwear modification	Modification to existing footwear with an intended therapeutic effect, e.g. pressure relief.
In-shoe orthosis/orthotic	Term used for device put inside the shoe to achieve pressure reduction or alteration in the function of the foot. Can be pre-fabricated or custom-made.
Liner	Synonym for insole.
Medical grade footwear	Footwear that meets the specific needs of a person. Can be either pre-fabricated (see “Pre-fabricated medical grade footwear”) or custom-made (see “Custom-made medical grade footwear”).
Metatarsal pad	Small pad placed proximal to the metatarsal head to relieve focal pressure and transfer load more proximally.
Metatarsal bar	Bar extending across part of or the entire forefoot placed proximal to the metatarsal heads to relieve focal pressures and transfer load more proximally.
Off-the-shelf footwear	Readily available footwear that has not been modified and has no intended therapeutic functions.
Orthopaedic footwear	Synonym for “Custom-made medical grade footwear”.
Pedorthic footwear	Synonym for “Medical grade footwear”. Can be either pre-fabricated (in that case synonym for “Pre-fabricated medical grade footwear”) or custom-made (in that case synonym for “Custom-made medical grade footwear”).
Pedorthic footwear modification	Synonym for “Footwear modification”.
Pre-fabricated medical grade footwear	Pre-fabricated footwear that meets the specific needs of a person, on the basis of footwear that provides extra depth, multiple width fittings and features designed to accommodate a broader range of foot types. Other features may include modified soles, fastenings and smooth internal linings. This type of footwear is usually available at specialty shoe shops.
Pre-fabricated insole	An “off-the-shelf” flat or contoured insole made without reference to the shape of the patient’s foot.
Shoe insert	Synonym for insole or in-shoe orthosis.
Shoe last	Last used to make footwear. The upper of the footwear is moulded or pulled over the last. The last shape defines the footwear shape including the outsole shape, heel pitch and toe spring. For off-the-shelf or pre-fabricated footwear generically generated lasts in different sizes are used.
Therapeutic footwear	Generic term for footwear that is designed to allow some form of treatment. May refer to both custom-made or pre-fabricated medical grade footwear.
Toe orthosis	Synonym for “In-shoe orthosis”, but specifically for the toe.

Several healthcare disciplines may be involved in the provision of footwear for people with diabetes. Having a common vocabulary is essential for clear communication. We propose to use the following definitions, obtained from [15, 16, 32, 33] and authors’ expert opinion

The first draft of this guideline was written by the first author (JvN), and then sent to two co-authors (PAL and PW) for critical review and expert opinion. A second draft incorporating consensus feedback from the three authors was sent to all authors for critical review and expert opinion feedback. The authors of this guideline, all (inter)national experts in the field of diabetic foot ulcer and footwear management, came from the following backgrounds: podiatric medicine (*n* = 5), podiatric surgery (*n* = 1), human movement science (*n* = 2), wound medicine (*n* = 2), pedorthics (*n* = 1), rehabilitation medicine (*n* = 1), endocrinology (*n* = 1), and vascular surgery (*n* = 1). A third draft incorporating feedback from all co-authors was written by the first author (JvN) and again sent to all co-authors for review. This process was repeated one more time, until consensus was reached from all authors, leading to the final version of the guideline, approved by all authors.

### Definitions for foot risk status

The purchase and wearing of appropriate footwear is an important process of care for all individuals with diabetes. This importance increases as the individual’s risk for developing a foot ulcer increases. Different classifications for foot risk status are used worldwide. For the purpose of this Australian footwear guideline we followed the classification provided in the Australian NHMRC guideline [31]:

(i) Low-risk of foot ulceration: people with no identifiable risk factors on foot screening (no peripheral neuropathy, peripheral artery disease, foot deformity, previous foot ulcer, or history of lower-extremity amputation).
(ii) Intermediate-risk of foot ulceration: people with only one risk factor on foot screening (either peripheral neuropathy, peripheral artery disease or foot deformity) and no previous foot ulcer or amputation.
(iii) High-risk of foot ulceration: people with two or three risk factors on foot screening (peripheral neuropathy, peripheral artery disease or foot deformity) or with a previous foot ulcer or amputation.

According to the NHMRC guideline, Aboriginal and Torres Strait Islander people with diabetes are considered to be at high-risk for foot ulceration, until the person’s level of risk is adequately assessed and confirmed otherwise [31].

To determine foot risk status, all people with diabetes should undergo at least a yearly foot screening by an appropriately trained registered healthcare professional with demonstrated competency [14, 31]. People with an intermediate- or high-risk foot status should be screened at least once every 3 to 6 months [14, 31]. In accordance with the NHMRC guideline, this should consist of screening for peripheral neuropathy (10 g monofilament sensitivity; vibration perception; neuropathy disability score), peripheral artery disease (palpation of peripheral pulses; ankle-brachial pressure index; toe-brachial pressure index), foot deformity (six point scale scoring small muscle wasting, Charcot foot deformity, bony prominence, prominent metatarsal head, hammer or claw toes and limited joint mobility), and assessment of a history of foot ulcer(s) or lower-extremity amputation [31]. The Australian Diabetes Society has published a video-example of such a foot examination [35].

### Structure of the guideline

This guideline consists of three parts and a discussion. Firstly, footwear recommendations and their rationale are provided for people at-risk of foot ulceration; These recommendations apply to people at low-, intermediate- or high-risk. Secondly, additional specific footwear recommendations and their rationale are provided for people at intermediate- or high-risk of foot ulceration. Thirdly, footwear and offloading recommendations for people with a diabetic foot ulcer are summarised. Finally, considerations on footwear provision, on education and adherence, on cultural and geographical differences, and on methodology and terminology are discussed.

## Results

This guideline contains 10 key recommendations to guide health professionals managing people with diabetes choosing the most appropriate footwear for the person’s specific foot risk needs (Table 2). The recommendations and their rationale are described separately in this section.

**Table 2:** Recommendations on footwear for people with diabetes

#	Recommendations
For all people at-risk of foot ulceration	
1	Advise people with diabetes to wear footwear that fits, protects and accommodates the shape of their feet
2	Advise people with diabetes to always wear socks within their footwear, in order to reduce shear and friction
3	Educate people with diabetes, their relatives and caregivers on the importance of wearing appropriate footwear to prevent foot ulceration
For people at intermediate- or high-risk of foot ulceration	
4	Instruct people with diabetes at intermediate-or high-risk of foot ulceration to obtain footwear from an appropriately trained professional to ensure it fits, protects and accommodates the shape of their feet
5	Motivate people with diabetes at intermediate- or high-risk of foot ulceration to wear their footwear at all times, both indoors and outdoors
6	Motivate people with diabetes at intermediate- or high-risk of foot ulceration (or their relatives and caregivers) to check their: a. footwear, each time before wearing, to ensure that there are no foreign objects in the footwear, or penetrating, the soles b. feet, each time their footwear is removed, to ensure that there are no signs of abnormal pressure, trauma or ulceration
7	For people with a foot deformity or pre-ulcerative lesion, consider prescribing medical grade footwear, which may include custom-made in-shoe orthoses or insoles
8	For people with a healed plantar foot ulcer, prescribe medical grade footwear with custom-made in-shoe orthoses or insoles with a demonstrated plantar pressure reducing effect at the high-risk areas
9	Review prescribed footwear every three months to ensure it still fits, protects, and supports the foot
For people with diabetic foot ulceration	
10	For people with a plantar diabetic foot ulcer, footwear is not specifically recommended for treatment; prescribe appropriate offloading devices to heal these ulcers

### Footwear for people with diabetes at-risk of foot ulceration

#### Recommendation 1:

Advise people with diabetes to wear footwear that fits, protects and accommodates the shape of their feet.

#### Rationale

**Fig. 1:**
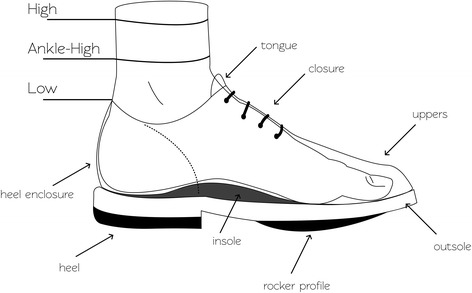
Footwear features. See Table 2 for a description of the requirements of these features

People with diabetes should wear footwear that fits, protects and accommodates the shape of their feet [14] (see Table 3 and Fig. 1). This includes having adequate length, width, and depth (and consequently adequate girth, i.e. adequate volume) [10, 15, 16, 32, 33]. A particular emphasis may need to be placed on the toe box of the shoe that should be consistent with the shape of the forefoot and toes of the person. An enclosed heel with a stabilising heel counter is recommended. Open-heel footwear can result in direct trauma injury to the heel and may require a person to claw their toes in order to keep the footwear fixed to their feet, further increasing the repetitive stress under their forefoot, and in turn the risk of ulceration. Adequate closure of the footwear is needed, to prevent the foot from sliding forwards and thus causing shear injury to the toes or plantar foot [36]. All features in Table 3 should be considered in combination, as their intended function is closely related and changes to one feature may affect other features and overall function [10, 15, 16, 32, 33].

**Table 3:** Requirements for footwear for people with diabetes

Feature	Requirements
Length	Inner length of the footwear should be 1–2 cm longer than the foot length as measured from heel to the longest toe when a person is standing. Adequate length needs to be confirmed when people are weight-bearing while wearing the footwear.
Depth	Depth should accommodate the toes to move freely without causing pressure at either the medial, lateral or the dorsal side.
Width	Width should equal the width of all parts of the foot. Width is good when the upper can be slightly bunched. The relation between forefoot and hindfoot is important, as accommodating a wide forefoot may result in the heel being too wide.
Height	Footwear height can be low, ankle-high, or high. High footwear provides more firmness, stability and reduces joint motion. The shaft of high footwear also contributes to forefoot pressure reduction. See further Table 3 for specific height requirements for people with a foot deformity.
Insole	The removable moulded insole can be pre-fabricated, adjusted or custom-made. The primary function of the insole is pressure redistribution. This is achieved via the principle of increasing the contact area between the foot and the insole, and the addition of corrective elements in the insole. Shock-absorbing, soft but sufficiently resilient and non-slippery materials should be used. See further Table 4 for the offloading effects of specific insole modifications.
Outsole	Rubber, plastic, and leather can all be used in construction of footwear outsoles, but rubber outsoles are thought to be superior. Outsoles can be supple, toughened or stiff. The shoe should not be more supple than the foot, or friction between foot and shoe will develop during push-off. See further Table 3 for specific outsole requirements for people with a foot deformity and Table 4 for the offloading effects of specific modifications.
Rocker profile	Rocker profiles have proven effectiveness in reducing plantar pressures, especially the forefoot. The rocker profile chosen depends on the affected joints and is determined by the apex position (pivot point) and the angle from the pivot point to the tip of the toe. For plantar pressure reduction of the metatarsophalangeal joints, the pivot point needs to be proximal to these joints. The rocker profile also impacts balance; the more proximally placed, the greater the balance disturbance. A person’s balance should therefore always be taken into account when deciding on the rocker profile.
Heel enclosure	An adequately fitting and enclosed heel is recommended, as open backed footwear or a heel enclosure that is too wide can result in injury and usually requires a person to claw their toes in order to keep them on. The heel counter needs to be free of edges protruding into the footwear.
Heel lift	The heel lift (or heel-forefoot difference, or pitch) should be generally 1.5–2 cm, and should not exceed 3 cm.
Closure	Adequate closure (or fixation) is needed to keep the foot from sliding forward. Closure should allow secure longer-term fastening and individual adjustment. Laces have long been considered the optimal choice; however, alternatives that are easier to use while still meeting these criteria are available as well, and innovative closures continue to be developed.
Uppers	The uppers consist of the ‘quarter’(hind- and midfoot) and ‘vamp’ (forefoot and toes). Uppers should be made from leather or a combination of materials (similar to sports shoes), with smooth inner lining made from a material that does not harden over time, with limited seams and preferably no seams in the vamp area as they reduce the ability of the leather to give. Uppers should be breathable and durable and have the ability to mould to deformities of the foot without resulting in pressure areas. Uppers can be supple, toughened or stiff. The vamp area should generally remain supple to accommodate the toes. See further Table 3 for specific requirements for the uppers (quarter) for people with a foot deformity.
Toe box	The part of the shoe that covers and protects the toes. This should be supple (unless specific requirements (e.g. for building professionals) require otherwise), and should accommodate the shape of the toes, to avoid any rubbing on the toes.

Features and requirements in this table are based on [10, 15, 16, 32, 33] and authors’ expert opinion; features are depicted in Fig. 1

People at low-risk of foot ulceration can usually be safely accommodated in a wide range of off-the-shelf footwear without specific requirements, provided the footwear is correctly fitted and appropriate for the activity to be undertaken [10, 15, 16, 32, 33]. For people at intermediate- or high-risk of foot ulceration, see recommendations 4-9. When new footwear is provided to a person with diabetes at low-risk of foot ulceration, advise them that a “wear-in” period may be needed where they slowly increase the number of hours per day the footwear is used, and that they should be extra vigilant of their foot health in this period.

#### Recommendation 2:

Advise people with diabetes to always wear socks within their footwear, in order to reduce shear and friction.

#### Rationale

People with diabetes should be advised to always wear socks within their footwear, to reduce shear and friction. Further, advise people with diabetes to wear socks made of mostly natural materials (to prevent undue moisture accumulation), that are seamless (to prevent undue repetitive stresses) and do not have elasticated cuffs (to prevent undue oedema).

#### Recommendation 3:

Educate people with diabetes, their relatives and caregivers on the importance of wearing appropriate footwear to prevent foot ulceration.

#### Rationale

People with diabetes, their relatives and caregivers should also be educated on the importance of appropriate footwear to prevent foot ulceration, and the importance of adherence to wearing it [14]. Also, the importance of annual foot screens to assess their risk of foot ulceration, and to return for further footwear advice if their foot risk status increases should be emphasised [14].

### Footwear for people with diabetes at intermediate- or high-risk of foot ulceration

#### Recommendation 4:

Instruct people with diabetes at intermediate-or high-risk of foot ulceration to obtain footwear from an appropriately trained professional to ensure it fits, protects and accommodates the shape of their feet.

#### Rationale

People with only one risk factor identified after foot screening (either peripheral neuropathy, peripheral artery disease, or foot deformity) are at intermediate-risk of foot ulceration, whereas people with two or three risk factors (peripheral neuropathy, peripheral artery disease or foot deformity) or with a previous foot ulcer or amputation are at high-risk of foot ulceration.

People who develop diabetic peripheral neuropathy lose protective sensation and their ability to feel pressure and pain. Thus, they may have a tendency to purchase poorly fitting footwear in an attempt to stimulate some sensory feedback [16, 37]. They also do not feel abnormally high repetitive stress (pressure or shear) caused by inappropriate footwear or walking barefoot and are more likely to develop pre-ulcerative lesions (e.g. callus or blisters) that subsequently lead to ulceration [9, 12]. People with peripheral artery disease are less likely to heal pre-ulcerative lesions or minor trauma due to inadequate perfusion, and as such need to avoid inappropriate footwear that may cause these situations. A foot deformity changes foot biomechanics and may lead to abnormally high repetitive stresses; high plantar pressure in particular increases the risk of foot ulceration and therefore needs to be accommodated. People with a previous foot ulcer are at high-risk of developing a new ulcer, with reported re-ulceration rates of 40–50% within the first 12 months after healing [6, 38]. Due to the high re-ulceration rates it is recommended that the term ‘diabetic foot remission’ is used with patients whose ulcer has healed, to highlight the need for ongoing vigilance to prevent ulcer recurrence [6, 39–41].

All people at intermediate- or high-risk of foot ulceration should be instructed to wear footwear that fits, protects and accommodates the shape of their foot (Table 3 and Fig. 1). Due to the complexities in accommodating the foot and the importance of preventing foot ulceration, people with diabetes should be instructed to obtain their footwear from an appropriately trained professional with demonstrated competencies in footwear fitting for this population, to ensure the footwear meets all requirements.

#### Recommendation 5:

Motivate people with diabetes at intermediate- or high-risk of foot ulceration to wear their footwear at all times, both indoors and outdoors.

#### Rationale

Because of their increased risk, people with diabetes at intermediate- or high-risk of foot ulceration should be motivated to wear their footwear at all times, both indoors and outdoors. When doing so, be aware that adherence to wearing footwear is significantly lower indoors compared to outdoors [30], while the majority of steps in these patient groups have been shown to be taken indoors [30, 42]. Depending on cultural preference, prescribing suitable footwear for outdoors and a second pair for indoors may be advisable. The indoor footwear should meet the same requirements with regard to adequacy of fit and offloading, but compromises might be made in the materials used in manufacture, as it is likely to experience less “wear-and-tear” compared to footwear used outdoor. See further the considerations on education and adherence.

#### Recommendation 6:

Motivate people with diabetes at intermediate- or high-risk of foot ulceration (or their relatives and caregivers) to check their:

- footwear, each time before wearing, to ensure that there are no foreign objects in the footwear or penetrating the soles.
- feet, each time their footwear is removed, to ensure that there are no signs of abnormal pressure, trauma or ulceration.

#### Rationale

People with peripheral neuropathy have lost the ability to feel pressure, pain or foreign objects. They, or their relatives and caregivers, need to be motivated to check their footwear each time before they are put on, to ensure that there are no foreign objects in the footwear or penetrating the soles. Furthermore, they should also check their feet each time their footwear is removed, to ensure that there are no signs of abnormal pressure, shear, trauma or ulceration. People should be advised to immediately seek help from an appropriately trained professional when their footwear is damaged or when signs of abnormal pressure, shear, trauma or ulceration on their feet are found.

#### Recommendation 7:

For people with a foot deformity or pre-ulcerative lesion, consider prescribing medical grade footwear, which may include custom-made in-shoe orthoses or insoles.

#### Rationale

When a foot deformity, pre-ulcerative lesion is present, off-the-shelf footwear is not likely to be appropriate. Prescribing medical grade footwear (pre-fabricated or custom-made; Table 1) needs to be considered, to accommodate the altered biomechanics. This medical-grade footwear may also include custom-made in-shoe orthoses or insoles. Depending on the foot deformity present or the location of the pre-ulcerative lesion, the footwear requirements algorithms for prescription (Table 4) and footwear modifications (Table 5) should be followed [25, 33]. The outsole, uppers and tongue can be “supple”, “toughened”, and “stiff” [33]. Toughened or stiff features facilitate the even distribution of forces exerted on the foot; unfortunately, no measurable definition of these is available [33].

**Table 4:** Specific footwear requirements for people with diabetes and a foot deformity

	Height	Outsole	Uppers (quarter)^b^	Tongue
Limited joint mobility	Low^a^	Toughened	Supple	Supple
Pes cavus	Ankle-high	Toughened	Toughened	Toughened^c^
Flexible flat foot with hallux valgus	High	Toughened	Toughened	Toughened^c^
Rigid flat foot with hallux valgus	Ankle-high	Toughened	Strong medial support	Toughened^c^
Charcot foot	High	Stiff	Toughened	Toughened^c^
Hallux or toe amputation	High	Stiff	Toughened	Toughened^c^
Forefoot amputation	High	Stiff	Stiff	Stiff

This table is based on [33]

^a^Unless a person has limited joint mobility in the ankle joint, in that case use ankle-high or high footwear

^b^The uppers consist of quarter and vamp, the requirements here concern the quarters, as the vamp typically needs to remain supple to accommodate the toes (see further Table 2)

^c^When a tongue is toughened, it should be padded as well

**Table 5:**
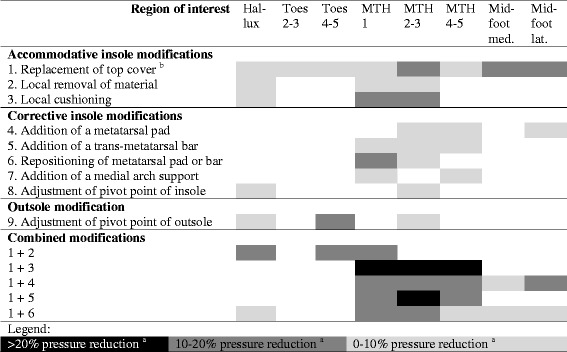
Plantar pressure reducing offloading effects of insole and footwear modifications

*Med*. medial, *Lat*. lateral. This table is based on [25]

For the blank cells in the matrix, there is either not enough information available, or pressure reduction was not statistically significant, or pressure increased; these modifications are therefore not recommended for these regions of interest

^a^Pressure reduction was significantly different from 0 (*p*-values < .05)

^b^Replacement with a new top cover of the same material

#### Recommendation 8:

For people with a healed plantar foot ulcer, prescribe medical grade footwear with custom-made in-shoe orthoses or insoles with a demonstrated plantar pressure relieving effect at the high-risk areas.

#### Rationale

For people with a healed plantar foot ulcer, off-the-shelf footwear is most unlikely to be sufficient. Medical grade footwear (pre-fabricated or custom-made; Table 1) with a demonstrated plantar pressure reducing effect at high-risk areas, including the previous ulcer location, needs to be prescribed. This medical-grade footwear should also include prescribed custom-made in-shoe orthoses or insoles to increase the plantar pressure reducing effect. Based on two recent randomised controlled trials, a ‘demonstrated plantar pressure reducing effect’ (combined effect of the new medical grade footwear with orthosis or insole) is defined as a > 30% reduction at the area of the highest plantar pressure in comparison to the same area in the patient’s current footwear, or a level below 200 kPa if measured with a validated and calibrated system with a sensor area of 1 cm^2^ [23, 24]. When such footwear is being worn by patients, the risk of re-ulceration is smaller [23]. Again depending on the location of the previous ulcer and presence (or absence) of a foot deformity and high-risk areas, follow the footwear requirements algorithms for prescription (Table 4) and footwear modifications (Table 5), with additional options provided by orthoses [25, 33]. The recommendation of prescribing footwear with a demonstrated plantar pressure reducing effect is in line with strong recommendations from the IWGDF guidelines [15], but has yet to be implemented widely in clinical practice in Australia. Different systems with different validity and reliability are available to quantify in-shoe plantar pressure [43]. We encourage services to invest in regular plantar pressure measurement protocols in daily clinical practice for people with diabetes and a healed plantar foot ulcer, and implementing the algorithms outlined in Tables 4 and 5.

#### Recommendation 9:

Review prescribed footwear every three months to ensure it still fits, protects, and supports the foot.

#### Rationale

Both the foot and the footwear change shape over time. Prescribed footwear, and custom-made orthoses or insoles, should be reviewed every three months to ensure it still fits, protects and supports the foot. This three-month interval is recommended based on the randomised controlled trial by Bus and colleagues, who used a three-month interval to ensure prescribed footwear remained appropriate, on expert opinion from seeing wear and tear in footwear in daily clinical practice, and aligns with the regular foot-screening interval for people at intermediate- or high-risk of foot ulceration as recommended in the NHMRC guideline [23, 31]. For people with a healed plantar foot ulcer who have been prescribed medical grade footwear with a demonstrated plantar pressure relieving effect, this effect still needs to be present for the footwear to be considered appropriate. Based on the findings from the trial by Bus and colleagues [23], ongoing research into its implementation in daily clinical practice and expert opinion, we suggest a three- to six-month interval for reviewing and demonstrating the plantar pressure relieving effect with validated equipment.

### Footwear for people with diabetic foot ulceration

#### Recommendation 10:

For people with a plantar diabetic foot ulcer, footwear is not specifically recommended for treatment; prescribe appropriate offloading devices to heal these ulcers.

#### Rationale

Footwear is not specifically recommended to treat a plantar diabetic foot ulcer in the IWGDF guidelines; in contrast offloading devices are recommended and necessary to heal these ulcers [15, 31]. We strongly recommend that any health professional treating a patient with a plantar diabetic foot ulcer ensures their patient has an appropriate offloading device. The most strongly recommended devices in the NHMRC guideline and IWGDF guidance documents are non-removable knee-high devices, such as a total contact cast or removable cast walker made irremovable [15, 31]. Only when knee-high devices are contraindicated or not tolerated by people with a diabetic foot ulcer should other offloading devices (such as forefoot offloading shoes and cast shoes), and lastly custom-made temporary footwear be considered [15].

Footwear for the unaffected foot of a person with a diabetic foot ulcer should follow the recommendations and criteria applied to people at high-risk of foot ulceration. Additionally, any height-difference caused by an offloading device may need to be corrected by adjusting the footwear of the unaffected leg. This can be achieved with internal footwear modifications or with external devices that are applied to the bottom of the shoe of the unaffected leg.

Prescribed footwear is needed once the ulcer is healed, again following the recommendations for people at high-risk of ulceration. When prescribed footwear cannot be made available immediately when the ulcer has healed, continuation in the offloading device meeting the offloading requirements is needed until the prescribed footwear becomes available (see further details under ‘considerations on footwear provision’).

## Discussion

This new 2017 Diabetic Foot Australia footwear guideline has updated the 2013 Australian footwear guideline to reflect the best available evidence from contemporary studies investigating footwear interventions, international guidelines and expert opinion. We have formulated 10 key recommendations to guide health professionals in selecting the most appropriate footwear to meet the specific foot risk needs of an individual with diabetes (Table 2), and provided the rationale behind these recommendations. In this discussion, we will add considerations on footwear provision, education and adherence, cultural and geographical differences, and methodology and terminology related to this guideline. These consideration provide further background with the recommendations, and discuss aspects relevant for implementation of the recommendations in daily clinical practice.

### Considerations on footwear provision

When providing footwear to a person with diabetes, ensure they know their foot risk status and confirm this via an evidence-based screening by an appropriately trained healthcare professional [31]. In addition to the foot screening, other factors that should be considered include the person’s gait pattern, activity levels, occupation, level of mobility, living situation, cultural beliefs, personal goals, and preferences. These factors may influence the possible options for appropriate footwear.

When providing footwear, measure the length, width, depth and girth of the foot the footwear needs to accommodate and ensure that the footwear follows the criteria in Tables 3 and 4. For length and width, we suggest at a minimum using a Brannock measuring device [44]. Although new scanning devices are becoming available to measure foot shape, we still suggest depth requires clinical assessment until accuracy of these devices can be independently quantified, taking into account that people with peripheral neuropathy cannot feel whether depth is accurate. Evaluate the shoe fit with the person in standing position, preferably at the end of the day to ensure that any developing oedema is taken into account. Further considerations in relation to oedema are footwear height (high footwear may have a compression function), outdoor temperature, and changes in oedema treatment.

The timing of footwear provision is important for any footwear that is not pre-fabricated. This becomes even more important when a person with diabetes at intermediate- or high-risk does not have appropriate footwear at a given moment. The longer a person needs to wait to receive appropriate footwear, the more steps they will take in inappropriate footwear, potentially increasing the repetitive stresses on the foot and in turn the risk for foot ulceration. Timing is most important for people with a recently healed plantar foot ulcer. Delivery of their prescribed footwear should be coordinated to a point as close to healing as possible. Ideally, the transition from an offloading device required to heal the ulcer to the preventative footwear is immediate. Any delay in this transition increases the risk of ulcer recurrence. When appropriate preventative footwear is not available for a person with a nearly healed foot ulcer, footwear prescription should be initiated before the ulcer is healed. Prescription can be initiated when foot shape (especially volume), structure and function are not expected to change during the healing process, and should take the manufacturing time-schedule into account. When prescribed footwear cannot be made available immediately when the ulcer has healed, continuation in the offloading device meeting the offloading requirements is needed until the prescribed footwear becomes available. Further, when new footwear is provided to a person with diabetes after healing a foot ulcer, advise them that a “wear-in” period may be needed where they alternate the new footwear with the offloading device that was required to heal the ulcer, and that they should be extra vigilant with checking of their foot health in this period.

In Australia, various state-based schemes are available that may provide financial assistance to people with diabetes who require medical-grade footwear. However, these schemes change over time and discussion of the specific schemes is outside the scope of this guideline.

### Considerations on education and adherence to wearing footwear

Early education on the importance of adequate footwear for foot health is important for all people with diabetes. This education needs to continue life-long, and needs to be expanded if a person’s level of risk of foot ulceration increases. The importance of footwear for people with diabetes should be discussed in the context of the individual’s foot risk status and health literacy [45]. Education should aim to increase people’s understanding of the requirements of their footwear to adequately fit, protect and accommodate their feet. This may also include, but is not limited to, education on proper donning of the footwear, the importance of wearing socks in footwear to reduce shear and friction, and explaining the risks to foot health of inappropriate footwear such as slippers and sandals, of narrow heels, of heels higher than 3 cm, and of pointy, flat or hard toe boxes. Education should further focus on motivating people with diabetes at intermediate- or high-risk of foot ulceration to wear their footwear at all times. Footwear can only be effective when it is worn, and adherence to wearing footwear is an important factor in foot ulcer prevention [17, 23, 26, 30].

Achieving better adherence is a challenge, and unfortunately we found no intervention studies on the effect of interventions that aim to increase footwear adherence in people with diabetes [17, 41]. However, we found a number of observational studies investigating reasons for (non-)adherence to footwear [26, 28, 45–49]. An improvement in walking has been described as the most important footwear-related characteristic affecting adherence, while the importance of cosmetic appearance and ease of use varies greatly between people [28, 48]. Rather than focussing on footwear characteristics, it is suggested in various studies that personal perceptions, values and experiences are more important factors to improve adherence [26, 28, 45–49]. A perceived benefit of footwear is associated with increased adherence to wearing the footwear [26], and conversely, a lack of understanding of the need for footwear hinders adherence [28]. Acceptance of the need for footwear is another important factor affecting adherence [28, 46, 47]. This does not only concern accepting the need for footwear, but also acceptance of the person’s underlying diabetic foot disease [28, 46, 47]. Footwear has been described as a “visible representation of the disease”, and people with diabetes at-risk of foot ulceration may choose to moderate their adherence to align with functional requirements and societal norms [28, 46, 47].

These personal values and experiences cannot be assessed using a standardised measurement device. Adequate communication between healthcare professionals and patients is needed to assess these perceptions [45, 50]. For this communication to be effective, it should be person-centred, not footwear-centred [45, 50]. Footwear is very personal, and this should be taken into account during education and communication to ensure maximum acceptance of and adherence with the footwear provided [28, 47, 50].

For people at intermediate- or high-risk of foot ulceration, the importance of adherence to wearing appropriate footwear both indoors and outdoors needs extra attention. People at risk of foot ulceration have been found to perform the majority of their total daily steps indoors [30, 42], while their adherence to wearing their footwear is significantly lower indoors compared to outdoors [30]. To improve adherence, people may need to be made aware of the greater repetitive stresses on their feet when at home resulting from the greater number of steps. It has also been suggested to provide separate footwear for indoor and outdoor use [30]. For people from cultures that may prefer not to wear ‘normal’ footwear indoors, it is suggested that health professionals consider providing indoor footwear that is manufactured to not look like ‘normal’ footwear, which may then be more acceptable to be worn indoors.

### Considerations on cultural and geographical differences

In this guideline, we describe features and criteria for footwear for people with diabetes, and specific recommendations based on a person’s foot ulcer risk following the NHMRC risk classification. Footwear is very personal and multiple other factors may need to be taken into account when providing footwear to a person with diabetes and ensuring this footwear is being used. We acknowledge the cultural differences in regard to footwear behaviour, specifically for Aboriginal and Torres Strait Islander people and from other diverse ethnic backgrounds. Furthermore, individuals in geographically rural and remote areas of Australia may have a limited range of footwear options available to them, and limited access to appropriately trained professionals. However, we decided not to provide specific recommendations for different cultures or for people living in rural and remote areas. The criteria and recommendations in this guideline are to be seen as the standards to be achieved, and these recommendations can be used by clinicians in their communications to discuss the footwear requirements for each person’s situation. Specific circumstances may require that a compromise is made to the recommendations, which then may be considered to be better than no footwear at all. However, in our opinion, offering deviations from the standards in this guideline, without supporting evidence and solely based on specific cultural or geographical backgrounds of people, does not align with offering equality of best practice care for all people and may increase the risk of foot ulceration and will weaken this guideline. Rather, we encourage healthcare professionals to use this guideline to discuss footwear requirements with people with diabetes, to try and achieve, if needed, a compromise that is optimal for the person’s situation that most closely aligns with the requirements and recommendations described in this guideline.

### Considerations on methodology and terminology

We have based this update of the 2013 guideline on contemporary evidence-based guidelines [10, 14, 15, 31], scientific evidence from systematic reviews [17–22], randomised controlled trials [23, 24], observational studies [9, 25–30, 32–34], and expert opinion, involving experts from eight different disciplines involved in the treatment of people with diabetic foot disease. However, this should not be looked upon as an evidence-based guideline, as we did not follow a specific guideline development methodology. Developing evidence-based guidelines is an extensive and costly process. With recent studies providing a much stronger evidence-base for footwear requirements for people with diabetes we felt that a new footwear guideline to update information in the NHMRC guideline [31] and the 2013 Australian practical guideline on footwear provision [16] was more important than waiting for completion of a full evidence-based guideline. Compared to the recommendations from the 2013 guidelines, some have not changed, and a number of new ones have been added. These include the need for health professionals to prescribe medical grade footwear that has demonstrated plantar pressure reducing effects at high-risk plantar areas for those people with a healed plantar foot ulcer, to review the adequacy of any prescribed footwear every three months, and to treat a plantar foot ulcer primarily with appropriate offloading devices. With this current document, healthcare professionals can immediately start to implement the new footwear evidence to begin to further reduce the large national burden of diabetic foot disease.

The specific footwear requirements are closely related to an individual’s foot risk status. This means that to provide people with diabetes with appropriate footwear, their foot risk status must be assessed first. We followed the classification as provided in the NHMRC guideline [31]. Other countries may use different risk classifications, and we advise healthcare professionals to ensure they use the guideline that is applicable in their own country with regard to foot risk status assessment. In this guideline, we did not separate between intermediate- and high-risk. The first reason for doing so was that some recommendations do not depend on foot risk status per se, but on the presence (or absence) of the specific risk factors of foot deformity or previously healed ulcer. To cover these differences, specific recommendations were needed that applied both to people at intermediate- and high-risk. Further, combining both groups while including specifically targeted recommendations also gives healthcare professionals from other countries the opportunity to match the recommendations in this guideline with their own country’s foot risk status classification system. Finally, the recommendations that did not target a specific risk factor were similar for people at intermediate- or high-risk, which means they could be combined.

As recommended in this guideline, people at intermediate- or high-risk of foot ulceration should be instructed to obtain their footwear from an appropriately trained professional with demonstrated competencies in footwear fitting for people with diabetes. We have not defined ‘appropriately trained’ or ‘demonstrated competencies’, as that was beyond the scope of the current document. However, as a minimum, we suggest an appropriately trained professional should be able to show documented evidence of their training and competency, and should meet the standards of their profession when such standards are available. This way, other healthcare professionals may confidently inform people with diabetes where to obtain their footwear.

The methodology followed to write this guideline does have some limitations. The first, not following a guideline development methodology, has been discussed above. A second is that no patient advocates were involved in its creation. This is a consequence of not following a specific guideline methodology, and we hope that this will be done in the next update of the NHMRC guideline [31]. A third is the limited evidence base with regard to the recommendations for people at low-risk of ulceration [17, 41]. These recommendations might be seen as “good practice statements”, a terminology used in official guideline development for recommendations that are predominantly based on expert opinion and standard of practice, when limited evidence is available [51]. As argued in other publications, it is hoped that researchers and healthcare providers combine efforts to build a stronger research evidence base for these recommendations [41]. Finally, we are unaware of cost-effectiveness information for any of the proposed footwear interventions [17], and thus no such specific information can be added to this guideline. However, a recent Australian cost-effectiveness analysis reported appropriately prescribed footwear as part of a suite of optimal diabetic foot care practice was always cheaper than standard care, and with the high costs associated with foot ulceration [1, 52, 53], it is likely that preventative footwear efforts in this regard will be cost-saving [41].

## Conclusion

Appropriate footwear is important for all people with diabetes, to prevent foot ulceration and reduce the burden of diabetic foot disease. This guideline contains 10 key recommendations to guide health professionals managing people with diabetes choosing the most appropriate footwear for the person’s specific foot risk needs. We hope that this guideline will be used to ensure that all Australians with diabetes have access to, and are provided with, appropriate footwear to meet their needs. This should improve footwear practice in Australia, and reduce the burden of diabetic foot disease for people and the nation.

## Abbreviations

IWGDF: International Working Group on the Diabetic Foot
NHMRC: National Health and Medical Research Council
